# Decoding of Human Movements Based on Deep Brain Local Field Potentials Using Ensemble Neural Networks

**DOI:** 10.1155/2017/5151895

**Published:** 2017-10-19

**Authors:** Mohammad S. Islam, Khondaker A. Mamun, Hai Deng

**Affiliations:** ^1^Department of Electrical and Computer Engineering, Florida International University, Miami, FL 33174, USA; ^2^AIMS Lab, Department of Computer Science and Engineering, United International University, Dhaka, Bangladesh; ^3^Nanjing University of Aeronautics and Astronautics, Nanjing, China

## Abstract

Decoding neural activities related to voluntary and involuntary movements is fundamental to understanding human brain motor circuits and neuromotor disorders and can lead to the development of neuromotor prosthetic devices for neurorehabilitation. This study explores using recorded deep brain local field potentials (LFPs) for robust movement decoding of Parkinson's disease (PD) and Dystonia patients. The LFP data from voluntary movement activities such as left and right hand index finger clicking were recorded from patients who underwent surgeries for implantation of deep brain stimulation electrodes. Movement-related LFP signal features were extracted by computing instantaneous power related to motor response in different neural frequency bands. An innovative neural network ensemble classifier has been proposed and developed for accurate prediction of finger movement and its forthcoming laterality. The ensemble classifier contains three base neural network classifiers, namely, feedforward, radial basis, and probabilistic neural networks. The majority voting rule is used to fuse the decisions of the three base classifiers to generate the final decision of the ensemble classifier. The overall decoding performance reaches a level of agreement (kappa value) at about 0.729 ± 0.16 for decoding movement from the resting state and about 0.671 ± 0.14 for decoding left and right visually cued movements.

## 1. Introduction

A fundamental function of the brain-machine interfaces (BMI) is to decode and interpret the recorded neural potentials to classify the patient's intentions or intended behaviors. Such information allows for a better understanding of neuronal circuit mechanisms and enables possible development of treatment methods for neurodegenerative disorders [1].

Deep brain stimulation (DBS) [2–4] is a functional neurosurgical procedure of implanting a miniature medical device to send electronic signals to certain parts of the brain such as subthalamic nucleus (STN) or globus pallidus interna (GPi) in Basal Ganglia (BG) for treatment of movement disorders such as Parkinson's disease (PD) or Dystonia. At the same time, DBS devices can be considered for BMI design and they are able to record the neurosignals called local field potentials (LFPs) [5–7] for body movement prediction or interpretation. Deep brain LFPs represent the aggregation activities of a large population of local synchronous neurons [5] and can provide neuronal information with better quality (i.e., high SNR) and greater stability over time compared with single-unit activity (SUA). The acquired LFPs from implanted DBS macroelectrodes can be used by researchers and clinicians to investigate on functioning of the Basal Ganglia in motor control [8] for better understanding and more effective treatments of movement disorders [9]. Deep brain LFPs reflect synchronized, subthreshold currents generated in the somata and dendrites of local neuronal elements [10] and they can be subdivided into a number of frequency bands including delta (0–3 Hz), theta (4–7 Hz), alpha (8–12 Hz), beta (13–32 Hz), gamma (31–200 Hz), and high-frequency (>200 Hz) [9] bands. During human body movements, the frequency of the LFP signals can be as high as 300 Hz [7] and is likely to vary due to a varied degree of behavioral and disease correlation. For example, in case when self-paced (voluntary), externally cued movements or any specified action is intended to be performed, the frequency-dependent event-related synchronization (ERS) and event-related desynchronization (ERD) can be found in various LFP bands recorded in bilateral STNs and/or GPIs [5, 10], which suggests that these oscillations may be related to the preparations of motor response.

With the analysis of intra-operative LFP recordings, it has been found that the frequencies of the synchronized oscillatory activities generally belong to one of two different bands for PD patients withdrawn from dopaminergic therapy [10]. The first band contains activity frequencies (3–12 Hz) of Parkinsonian rest and action tremor, but the signal in this band is neither consistent nor a strong feature of LFPs. However, the second band, called beta band (13–32 Hz), is the frequency range representative of LFP oscillations. This band is antikinetic in nature and is manifested in single-unit activity [10]. Furthermore, for PD patients, the improvement in bradykinesia and rigidity with the subsequent dopaminergic therapy was shown to be correlated to the signal magnitude change in the beta band [9]. However, for PD patients, the oscillatory characteristics of beta frequency band are augmented to such an extent that they dominate over motor commands used for initializing voluntary movements, leading to movement disorders [13]. The most consistent of beta band activities can be found in the untreated, hypodopaminergic Parkinsonian state [14–16]. Recent study also substantiated that the strong signal components in beta frequency band were observed in LFPs recorded from the GPI of PD patients, whereas, for Dystonia patients, the signal in the same frequency band was much less salient [9]. For Essential Tremor (ET), the tremor signals are consistently in the frequency range of 8–27 Hz. For cervical Dystonia, the frequency ranges of 4–10, 11–30, and 65–85 Hz of LFPs are highly correlated to sternocleidomastoid muscle EMG signal frequencies [9]. In addition, ERD in beta band (10–24 Hz) was observed during human movement initiation process and ERS during cessation of movement [9]. At rest and during “OFF” medication Parkinsonian state, alpha (8–12) Hz and beta (13–32) Hz oscillatory activities dominate in the LFP frequency spectra, while they are drastically reduced during “ON” medication state [7]. Moreover, during “OFF” levodopa, the activity in gamma band increases bilaterally during active movement [9] and high-frequency oscillations (HFO) (300–350 Hz) may heighten. In addition, it was also reported that, during “ON” and “OFF' medication states in PD, the extent of power in the frequency band of 4–10 Hz is lower in contrast to Dystonia patients [9]. Although the oscillations in gamma band (>70 Hz) in LFPs that is correlated to human movement (prokinetic) were suppressed [13] or absent in PD patients, during the “ON” medication state, the synchronized oscillatory activity may occur in the STNs and GPIs. Although the evidence suggests that these frequency activities would increase when the body changes from rest to movement, the activities above 65 Hz appear to be an unreliable LFP feature for PD patients [10].

Basal Ganglia STNs activity can be modulated, while patient intends to perform a specified action or watches visual images of movements [17]. Such intended movements are responsible for generating ERS and ERD in Basal Ganglia which are similar in frequency and time to those during actual voluntary movement [1]. Although the differences in the midst of contra- and ipsi-lateral movement-related oscillatory changes in the STNs have been unknown, some studies suggest that there may not be substantial differences. However, it was also reported recently that, during wrist movement tasks, both contra- and ipsi-lateral ERS were observed in the gamma frequency band [7] but event-related desynchronization (ERD) was found in the low-beta frequency band (~10–24 Hz) [9].

Therefore, multiple frequency-dependent oscillations in motor cortex and BG are directly related to the process of action making, preparations, executions, and imaginations of movements [7]. Recent experimental results showed that, based on distinct oscillations of LFPs, self-paced hand movements can be predicted using a pattern recognition algorithm [18]. The result indicates that LFP activity is directly or indirectly involved in the process of motor preparation. In addition, it is found that the LFPs can be used to infer substantial information about specific types of arm movement parameters such as distance, speed, and directions for motor disorder patients [19, 20]. A recent study showed that movement in eight directions can be decoded with the best recognition rate of up to 92% using the spatial patterns of LFPs in premotor and primary motor areas [19].

Some studies have been conducted to find the coherence and causality between cortex and hand movement. In one study, it was found that noteworthy coherence only exists between the human sensorimotor cortex and contralateral hand and forearm muscles. However, no existence of coherence was found in sensorimotor cortices or any ipsi-lateral hand and forearm muscle [21]. In another study, it was shown that voluntary movement can be decoded up to 76.0 ± 3.1% using causal strength of LFP signal features computed on neural synchronization of bilateral STNs or GPIs and utilizing bivariate Granger Causality [1]. Additionally, it was found that left and right hand movements are associated with different spatiotemporal patterns of movement-related synchronization and de-synchronization [22]. Therefore, motor control or bilateral coordination can be predicted by decoding movement intention from Basal Ganglia neural activities for left and right hands [1, 7, 12]. These research findings have further demonstrated that LFPs during onset of movement contain supportive information that may advance our knowledge towards reliable movement decoding strategies for neuroprosthetic device developments, diagnostic assessments, and possible treatment of some chronic neurological disorders. For instance, early prediction of onset of tremor of PD patients may provide the possibility of constructing an adaptive therapeutic intervention mechanism in using DBS for optimal neuromodulation effects [3].

Hence, the prediction and classification of human body movements can be achieved by decoding the recorded BG LFP signals using pattern recognition algorithms. In this paper, we have developed an innovative neural network (NN) based ensemble classifier for effectively decoding the LFP signals recorded from sequential occurrence of movements and identifying whether the movement is left- or right-sided visually cued in an automated and systematic fashion.

Artificial neural networks (ANNs) [23] are one of the most effective and commonly used machine learning algorithms. However, different types of ANN algorithms possess various advantages and disadvantages in classification. For instance, the FBANN, that is, multi-layer perceptron (MLP), is relatively efficient in optimization or classification with limited training data but tends to be stuck in the local minima and provides less satisfactory classification results [24]. On the other hand, RBFNN could find the global minimum [25] but requires much larger dataset to train. Alternatively, PNN, derived from the Bayes rule and kernel Fisher discriminant, is more accurate than MLP networks and insensitive to outliers in training data [26]. However, PNN needs more training data and is slower than MLP networks in classification. Therefore, it is highly preferable if we can design an ensemble classifier that uses all of the neural networks as the base classifiers for their collective advantages. The ensemble classifier would contain all the advantages of the above-mentioned networks for better activity decoding and classification using LFP dataset. Also, to get robust and consistent movement in decoding performance, we develop a decision fusion algorithm based on the majority voting strategy to combine the classification results from three individual neural networks. The majority voting is simple, intuitive, and effective ensemble approach for improving classification performance [27, 28]. Recently, it has been shown that when seven base classifiers were used in five different ensemble strategies, including majority voting, Bayesian, logistic regression, fuzzy integral, and neural network, the majority voting strategy proved to be as effective as any other algorithm in improving overall classification performance for the dataset provided [28]. We believe that identifying visually cued voluntary movements by decoding oscillatory characteristics of LFP activity may provide ways of developing more advanced neural interface systems such as BCIs and BMIs to enhance our understandings of the underlying process of movements and its important implications in STNs or GPIs for controlling movement activities.

## 2. Experimental Framework and Data Acquisition (DAQ) System

The LFP datasets used in training and testing for movement recognition were recorded through the DBS devices from the patients with Parkinson's diseases (PD) or Dystonia. The circumstances of the data acquisition are described in detail in this section.

### 2.1. Patient Details

In this work, a total of twelve Parkinson's disease or Dystonia patients (7 males and 5 females) with their ages ranging between 23 and 72 years (49.6 ± 13.9, mean ± 1SD) were recruited. Each patient underwent bilateral implantation of deep brain stimulation (DBS) electrodes in the STN or GPI for therapeutic stimulation to provide the LFP signals for recording. Their disease-suffering durations were between 3 and 38 years (14.8 ± 10.3, mean ± 1SD). The corresponding demographics are summarized in Table 1. The LFP data collection was approved by the local research ethics board at Oxford University. All participants provided written consent prior to this study.

### 2.2. Deep Brain Stimulation (DBS) Electrode Setup

The DBS macroelectrode (model: 3387, manufacturer: Medtronic Neurological Division, Minneapolis, USA) was implanted bilaterally in the left and right STNs or GPIs for treatment of the patients with Parkinson's disease or Dystonia. The macroelectrode consists of four platinum-iridium cylindrical surfaces (diameter: 1.27 mm, length: 1.5 mm, and center to center spacing: 2 mm; contact-0 is the most caudal and contact-3 is the most rostral). Macroelectrodes were inserted after STN and had been identified by using ventriculography and pre-operative magnetic resonance imaging (MRI). Stimulation spots were chosen as the electrode positions, where lessening in Parkinsonian symptoms occurred during intra-operative electrical stimulation and the matching is confirmed by examining the post-operative MRI scan or the fused images of pre-implantation MRI with post-implantation CT.

### 2.3. Movement Activities of the Patients

**Figure 1:**
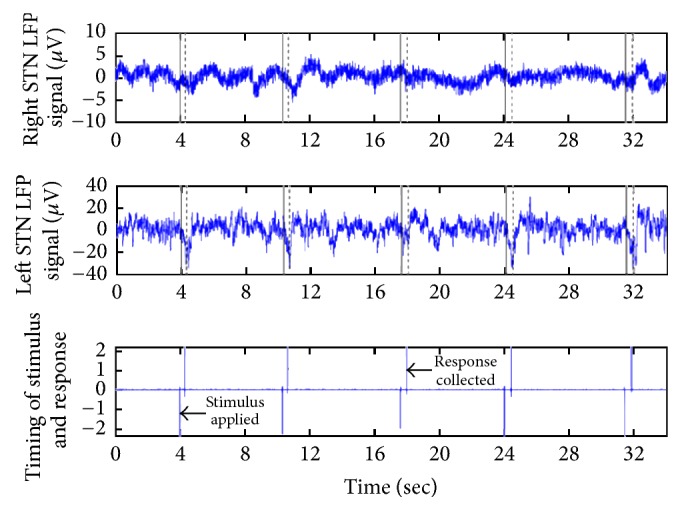
Recorded LFP signal from bilateral STNs with chronological visual stimulus applied to the patient. Time of stimulus is presented in solid lines and subsequent motor activity is presented in dotted lines.

During LFP recording from STNs (Figure 1) or GPIs, all subjects were instructed to do a finger pressing task in a random order with a short resting period between tasks. Each subject was seated 60 cm (approx.) away from the experimental computer screen. After that, prior to each motor task, they were instructed to keep their left or right index fingers on the distinct keys on the left or right standard keyboard. In addition, all the patients were asked to look at a 10 mm cross that was repetitively displayed in the center of the screen and letter A (height: 8 mm; width: 7 mm) on the screen for the duration of 400 ms instantly to the left or right central cross. It was the indication signal to the patients to move the finger. The interval of cues and laterality were provided randomly in the experiment.

### 2.4. LFP Signal Acquisition from Patients

The LFP signals of twelve patients were recorded at STNs and GPIs for 4–6 days via externalized electrode leads post-operatively after all the patients had been kept “OFF” medication overnight and high-frequency stimulation pulses were completely turned “OFF.” Using MRI, the DBS lead contacts at STNs or GPIs to record LFP signals on both sides were confirmed. Three adjacent pairs consisting of 4 contacts named 0, 1, 2, and 3, respectively (pair positions are 0-1, 1-2, and 2-3), were used to record LFPs in the bipolar signal form and bilaterally. Usually, the bipolar configuration was used to provide “common mode rejection” to far-field activity signals against common mode noise contamination. If DBS stimulation and activity recording are conducted simultaneously, the LFP signal recording can be interfered by the DBS stimulation pulses, leading to inaccurate recording and decoding results. In this experimental setup, we recorded the LFP signals well before the stimulation started to avoid any possible interference of the simulation pulses to activity recording. DBS macroelectrode pairs were chosen for better therapeutic effects and anatomical structures. After that, the segments of the recorded signal containing erroneous, premature, or no responses were deliberately discarded from the datasets. The number of trials had to be kept at minimum to minimize the stress during the experiment imposed on the PD/Dystonia patients. In the experimental session, 114 ± 43.6 trials (mean ± 1SD) consisting of minimum 56 and maximum 202 trials across all subjects were employed in the movement decoding process. In addition, for most of the patients, the number of trials is unbalanced for each class. The average number of trials of each class is 58.2 ± 23.6 (mean ± 1SD) with a minimum of 25 trials and maximum of 113 trials and the average difference between the classes across all the subjects is 14.2%  ± 19.0 trials (with a minimum of 1.2% and a maximum of 57.6%). The DBS surgery was only warranted if the patient had exhibited motion-related dysfunction in postural control, gait, and locomotion in addition to usual motor symptoms such as tremor, rigidity, and bradykinesia. Under these circumstances, there will be always challenges with the amount of data with sufficient neuronal information to be collected; therefore to develop an analysis method that does not rely on a large number of trials is of paramount importance. However, for avoiding rapid repetitive movements and obtaining valid ranges of inter-movement data, the LFP signals obtained outside the time range between 1 s and 5 s during a movement were excluded from the datasets. The contact pair (from bipolar mode: 0-1, 1-2, and 2-3) in the Basal Ganglia were chosen for analysis and showed greatest percentage of beta (*β*) band (13–32 Hz) modulation due to the movement in contrast to the amplitude of *β* modulation during the baseline activity period occurring 1-2 seconds before the onset of motor response. The LFP information obtained from the available contact pairs of each electrode would be highly correlated and therefore only one contact pair of each electrode was used for data recording and analysis. In the recording scheme, CED 1902 amplifiers (×10,000) were employed to amplify the initial signals recorded at the DBS contacts. With tripolar configurations (active-common-reference), surface EMGs were recorded using disposable adhesive Ag/AgCl electrodes (H27P, Kendall-LTP, MA, USA). Based on the recorded EMGs from the index finger, the onset of motor response and other voluntary and involuntary movements were determined by timing of the key presses as registration of motor response. The movement-related artifacts due to equipment lead were carefully identified and the recordings containing excessive noises were excluded from analysis. Contaminated trials with artifact were also removed. In addition, noise of the recorded data related to patients' movement were avoided as much as possible by instructing patients to stay in steady condition during each session of recordings. In the recorded EMGs, rest and movement conditions were defined as follows: “rest” is defined as no or little hypertonic bursts, “voluntary movements” are defined as regular pulses with a duration of tens of milliseconds, and “uncontrolled contractions” are defined as phasic spasm over seconds. The initial signals were amplified using isolated CED 1902 amplifiers (×10,000 for LFPs and ×1000 for EMGs), low-pass filtered with a cut-off frequency of 500 Hz, and then digitized using 12-bit CED 1401 mark II with a sampling rate of 2000 Hz. Subsequently, a custom written program in SPIKE 2 (Cambridge Electronic Design (CED), Cambridge, UK) software was used for recording, online monitoring, and storing the digitized data in the hard drive. Variations of instantaneous magnitude and frequency for both LFPs and EMGs were compared to find correlations between them during movement activities.

### 2.5. Preprocessing of STN's LFP Signals

For removing high-frequency noise and artifacts, a low-pass type-I Chebyshev filter (zero phase shifting and cut-off frequency 90 Hz) was applied to the STN's LFP signals. A notch filter at 50 Hz was further applied to the processed signals to remove the single-frequency noise associated with the power supplies. Then the LFP datasets were digitally resampled at 256 Hz prior to feature extraction and classification processing.

## 3. Methodology of Feature Extraction of LFP Signals Using Wavelet Packet Transform (WPT) and Hilbert Transform (HT)

To carry out the identification of finger movements from the LFP data, we used wavelet packet transform (WPT) and Hilbert Transform (HT) to extract the LFP signal features from different frequency bands in the frequency range from 0 to 90 Hz. For non-stationary biosignals such as LFPs, WPT is a better alternative as a data analysis tool than STFT or standard DWT in extracting relevant signal features for pattern recognition in the time-frequency domain [29].

WPT can decompose both approximation and detail spaces into further subbands with functionally distinct scales in a balanced binary tree and has ability to localize any specific information of interest as compared to DWT [30, 31]. In carrying out the WPT at decomposition scale of 5, the discrete Meyer wavelet (demy) was selected and applied to the LFP data to generate different multi-resolution coefficients. The WPT coefficients are obtained by recursively filtering out the coefficients generated in the previous stage with lower resolutions to compute the WPT coefficients at current scale.

**Figure 2:**
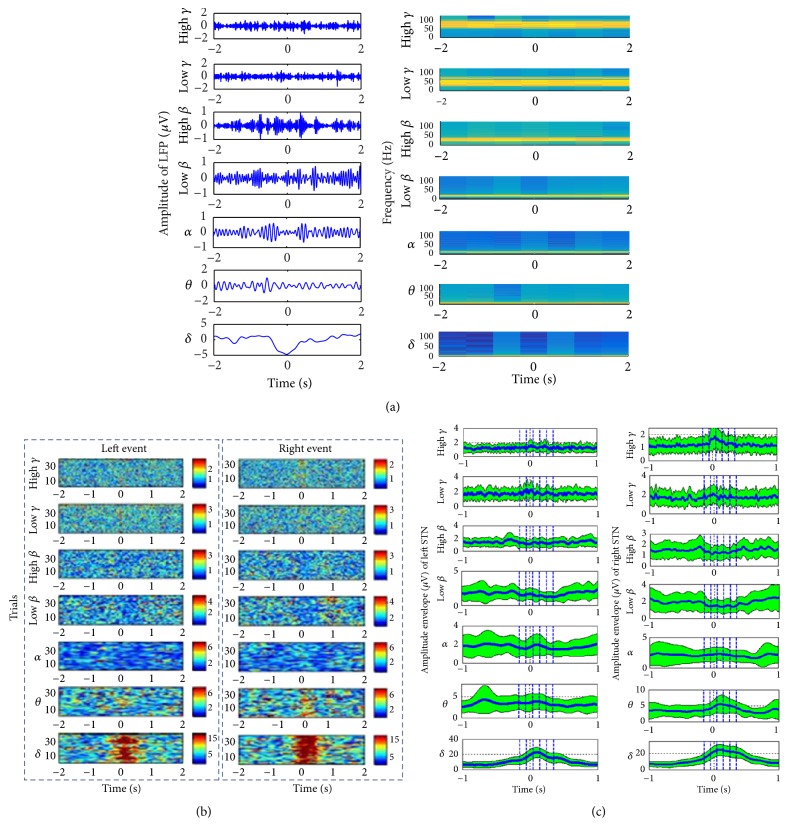
(a) Instantaneous amplitude (left) and spectrogram (right) of the right-sided STNs LFP for all frequency bands of patient #1 in a 4-second window centered at the time of response and visually cued left finger clicking events; the extracted frequency bands of LFP signal are delta (0–4 Hz), *θ* (4–8 Hz), *α* (8–12 Hz), low *β* (13–20 Hz), high *β* (20–32 Hz), low *γ* (32–60 HZ), and high *γ* (60–90 Hz), respectively, where high *γ* band [11] is not the same as the conventional high gamma band (80–200 Hz). (b) The instantaneous magnitude of different bands computed using Hilbert Transform (HT) for all trials of patient #1 during left and right finger visual cued clicking events obtained from deep brain's left STN LFPs (motor responses situated at the center of each time scale) [12]. (c) The average instantaneous magnitude (blue line) and standard deviation (SD) (green shadow area) acquired from STN LFPs of each component for patient #1 and visual cued left and right finger clicking events within 2 s time window. For each frequency band, LFP signal features were defined with average amplitude in five segments (area covered by dotted line.)

After completion of the WPT processing, we segmented a 4-second time window from each frequency band for LFP's left and right clicking event tasks at each motor response registration (Figures 2(a) and 2(b)). Likewise, we can segment the resting activity into a total of 2-second time windows during each stimulus registration. The signal envelope in each frequency band of the reconstructed signal was computed by using the Hilbert Transform (HT) [32] and the signal features were extracted based on the power of each frequency band. From Figure 2(c), it can be seen that event-related synchronization and desynchronization happened in all frequency bands but visible amplitude decrement was found in *β* band at the left and right STNs or GPIs. However, at the event onset, the signal amplitude in the *δ* band was quite large compared to those in other bands.

For generating the classification features, instantaneous power was computed by averaging the amplitudes of the defined windows in each frequency band. The window length was either 100 ms or 50 ms and its center locations were varied from −500 ms to +500 ms. Ultimately, based on the left and right visually cued movements and the oscillatory characteristics of STN's or GPI's LFP signal due to mean energy increment (synchronization) or reduction (desynchronization), the average amplitudes of five consecutive windows (from −150 ms to 350 ms) of length of 100 ms were chosen as the desired period of interest for feature extraction (Figure 2(c)). Similarly, feature extractions were conducted for the resting state. The five windows with a window size of 100 ms from −750 ms to −250 ms were selected to extract features for resting condition (prior to the stimulus applied) of the patients.

Finally, for each patient in each frequency band, vectors of total seventy bilateral features (2 sides × 7 bands × 5 points in time) at contra- and ipsi-lateral STNs or GPIs were extracted for decoding voluntary movement and resting activity.

## 4. Design of the Neural Network Based Ensemble Classifiers for LFP Data Recognition

**Figure 3:**
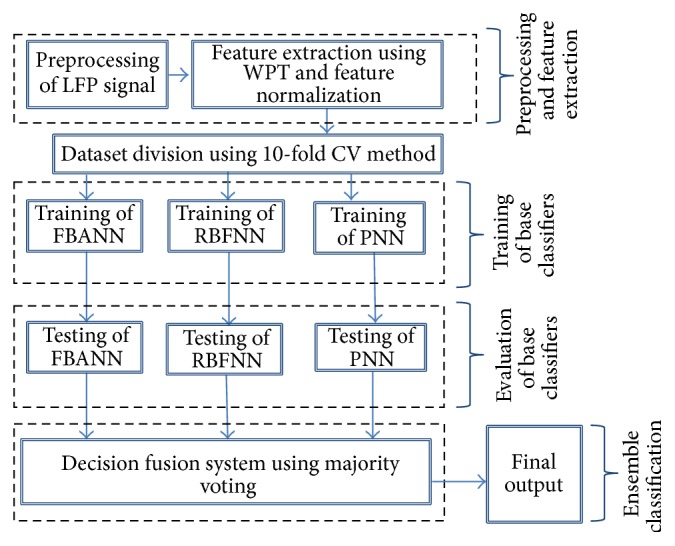
Proposed architecture of the ensemble classifier for training, testing, and evaluation.

The objective of the work is firstly to detect if finger movement has happened by decoding deep brain-recorded LFP signals and, if so, subsequently to determine the laterality of that movement. The decoding process, which is actually a two-step three-class classification, consists of LFP data acquisition and preprocessing part, the signal feature extraction part using WPT and HT, and the ensemble classifier that includes three base neural network classifiers and a fusion decision system. The structure of the ensemble classifier for the decoding process is shown in Figure 3.

**Figure 4:**
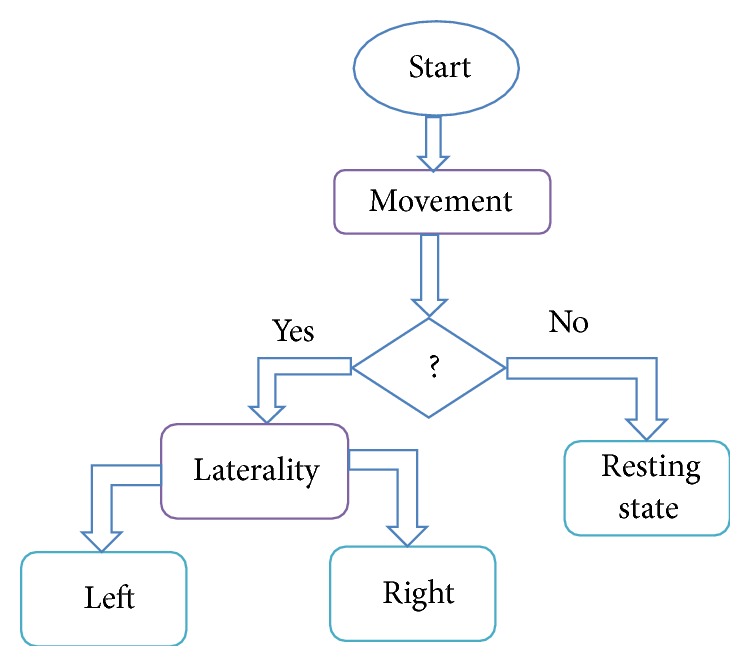
Movement detection and its subsequent laterality decoding process using bilateral deep brain's (STNs or GPIs) LFP signal.

The proposed overall decoding process using the ensemble classifier in Figure 3 is illustrated in the state diagram in Figure 4. The three base neural network classifiers used in the ensemble classifier will be briefly reviewed and the decision fusion rules and the performance evaluation approaches will be introduced in the rest of the section.

### 4.1. Three Base Neural Network Classifiers

Three different neural networks that will be used as the base classifiers to form the proposed ensemble classifier will be discussed very briefly in this section.

### 4.2. Feedforward Backpropagation Artificial Neural Network (FBANN)/Radial Basis Function Neural Network (RBFNN)/Probabilistic Neural Network (PNN)

The FBANN [33, 34] was originally designed and trained based on the steepest descent training algorithm. The FBANN network's overall output, *∅*, with an input vector* Xq* is computed based on the following equation:

(1) $$\begin{array}{c} \emptyset =f\left(\;\overset{n}{\underset{p=1}{\sum }}w_{p}^{2}f\left(\;\overset{m}{\underset{q=1}{\sum }}W_{pq}^{1}X_{q}+b_{p}^{1}\;\right)+b^{2}\;\right), \end{array}$$

where *W*_*pq*_ (*q* = 1,2,…, *m*; *p* = 1,2,…, *n*) are the connection weights, *n* is the total number of hidden nodes, and *m* is the total number of the input nodes used to fully connect with the hidden layers. Also, *f* is the nonlinear activation function. On the other hand, unlike FBANN, the RBFNN consists of an input layer, a hidden layer embedded with a nonlinear RBN activation function, and an output layer [35]. The PNN [26] consisting of input, pattern, and decision layers is capable of performing classification tasks for multi-class problems. The decision layer classifies the patterns of the output of the summation layer according to Bayes optimal decision rule.

### 4.3. Decision Fusion Rule

To obtain an unbiased decision on movement identification, we will use the majority voting-based ensemble classifier for decision fusion processing. For ensemble classifier, the decisions of the base classifiers are assumed to be autonomous and the final decisions are derived from a mixture of all base system's decisions [36]. Inherently, in the plurality voting strategy, the ensemble decision picks class *w*_*j*_, if there is

(2) $$\begin{array}{c} \overset{T}{\underset{t=1}{\sum }}d_{t,j}=\overset{C}{\underset{j=1}{max}}\overset{T}{\underset{t=1}{\sum }}d_{t,j}, \end{array}$$

where *d*_*t*,*j*_ is the decision taken by *t*th base classifier (*t* = 1,2,…, *T* and *j* = 1,…, *C*); *C* is the number of classes and *T* is the total number of base classifiers used. For plurality voting, if *t*th classifier predicts class of *w*_*j*_, then *d*_*t*,*j*_ = 1 or 0 for other cases.

In this work, because three base classifiers FBANN, RBFNN, and PNN are used, the majority rule dictates that any two or three base classifiers with the same decision would decide on the acceptance or rejection of the input data as the final decision.

### 4.4. Performance Evaluation

For classification purpose, a maximum of 28,280 data points from the patients were used for decoding movement versus rest activities. On the other hand, a maximum of 21,210 data points were employed to decode left- and right-sided visually cued movement activities. In our work, we used bootstrap resampling technique (i.e., random samples were chosen with replacement) in selecting movement and resting datasets of the patients. The corresponding number of trials for movement from each patient is shown in Table 2. Bootstrap is a useful statistical method widely used for classification performance assessment [37]. For a class *w*_*j*_, if the training set is *X*_*N*_*i*__^*j*^ = {*x*_1_^*j*^, *x*_2_^*j*^, *x*_3_^*j*^,…, *x*_*N*_*i*__^*j*^}, one can construct the bootstrap samples as follows. Firstly, one sample, *x*_*k*_0__^*j*^ from *X*_*N*_*i*__^*j*^, is randomly selected, and the *r* nearest neighbor samples (*x*_*k*_1__^*j*^, *x*_*k*_2__^*j*^,…, *x*_*k*_*r*__^*j*^) from *x*_*k*_0__^*j*^ are found based on the Euclidean distance. Then, the bootstrap samples are generated using *x*_*k*_0__^*b*^ = ∑_*i*=0_^*r*^*w*_*i*_*x*_*k*_*i*__^*j*^, where *w*_*i*_ = *c*_*i*_/∑_*d*=1_^*r*^*c*_*d*_; ∑_*r*_*w*_*i*_ = 1; and *r* ≥ *c*_*d*_ ≥ 0 [38]. Gaussian distribution (GD) used to choose *c*_*d*_ and the whole process is repeated until the whole *N*_*i*_ are selected.

To evaluate the overall classification performance of the proposed ensemble classifier, we used 10-fold cross-validation (CV) method to carry out the evaluation. For each design set, CV error was computed according to the following formula:

(3) $$\begin{array}{c} \text{CV error}=\frac{1}{N}\overset{N}{\underset{p=0}{\sum }}{\left[\;d_{p}\left(\;n\;\right)-y_{p}\left(\;n\;\right)\;\right]}^{2}, \end{array}$$

where *N* denotes the total number of samples and *d*_*p*_(*n*) is the desired output; *y*_*p*_(*n*) is the classifier's output for each test set and *n* denotes the number of conducted epochs. The design sets with the lowest error were considered for the base classifier learning and training. The threshold selection methods for all three base classifiers and the ensemble classifier are summarized in Table 3. Also, the pseudocode for proposed decision fusion algorithm for classification of movement or resting and left or right finger movement activities are listed in Algorithm 1.

The performance of the proposed ensemble classifier for movement detection and classification was evaluated by using several standard metrics such as cross-validated classification accuracy (CVCA), detection rate (DR), specificity (Table 4), *F*-measure, TPR, FPR, FNR, kappa, and AUC values. These performance metrics are derived from the standard contingency table based on four commonly used measures (TP/FP/TN/FN) that are commonly adopted in evaluating medical decision systems.

In the contingency table, true positive (TP) is the correct classification rate of the LFP signal generated from the movement state or left movement. True negative (TN) is the correct classification rate of the LFP signal generated from the resting state or right movement. However, false positive (FP) represents the classification rate of the LFP signals as movement or left movement, while they are actually resting state or right movement, respectively. False negative (FN) is the classification rate of the LFP signals as the resting state or right movement when the actual state is movement or left movement.

To obtain highest degree of desirability among the base classifiers to detect movement and resting activity, we have computed unified desirability measures using the following:

(4) Desirability1=meanprecisionstdprecision ×meansensitivitystdsensitivity ×meanspecificitystdspecificity ,Desirability2=meangmean-1stdgmean-1×meangmean-2stdgmean-2×meanF-measurestdF-Measure,Desirability=Desirability1×Desirability26.

Furthermore, to gauge the correctness of the classifier, we computed Mathew's correlation coefficient (MCC), as shown in (5). MCC in essence is a correlation coefficient between the observed and the predicted binary classification outcomes.

(5) MCC=TP×TN−FP×FNTP+FPTP+FNTN+FPTN+FN.

A value of +1 in (5) represents a perfect prediction; a value of 0 represents no better than random prediction and −1 indicates a total disagreement between the prediction and the truth. AUC is the area under the receiver operating characteristic (ROC) curve which is a useful measure in evaluating the performances of binary classification methods [39]. The AUC is defined as follows:

(6) $$\begin{array}{c} \text{AUC}=\frac{1}{2}\left[\;\frac{\text{TP}}{\text{TP}+\text{FN}}+\frac{\text{TN}}{\text{TN}+\text{FP}}\;\right]. \end{array}$$

For the sake of convenience and simplicity in comparison, we can compute the AUC values only instead of generating ROC curves, since they would be relatively tedious with the large number of datasets.

Alternatively, to obtain the inflated and more intuitive measure of the performance from the unbalanced datasets of the PD and Dystonia patients, we can use the following balanced accuracy (BACC) [40]:

(7) $$\begin{array}{c} \text{BACC}=\frac{1}{2}\left(\;\text{TPR}+\text{TNR}\;\right). \end{array}$$

To further measure the agreement between the predicted and desired classification results in the presence of unbalanced datasets, one can use Cohen's kappa coefficient as the agreement metric [41].

The kappa coefficient (*κ*) is estimated using the following equation:

(8) $$\begin{array}{c} \kappa =\frac{p_{0}-p_{e}}{1-p_{e}}, \end{array}$$

where *p*_0_ and *p*_*e*_ denote the classification accuracy and the expected agreement of chance, respectively, and these parameters can be calculated from the confusion matrix obtained from the proposed classifier.

If all values of *κ* within the 95% confidence interval (CI) around the mean are above 0 (*κ* ± 1.96 × *φ*(*κ*) > 0, where *φ*(*κ*) is the standard error), then the average kappa value is above the chance value. The standard error function, *φ*(*κ*), which is measuring the disagreement, is defined as

(9) $$\begin{array}{c} \varphi \left(\;\kappa \;\right)=\frac{\sqrt{P_{e}+P_{e}^{2}-\sum_{i}\left[n_{i+}n_{+i}\left(n_{i+}+n_{+i}\right)\right]}}{\left(1-p_{e}\right)\sqrt{N}}, \end{array}$$

where *n*_+*i*_ and *n*_*i*+_ are the marginal column and rows sums, respectively, and *N* is the total number of trials.

## 5. Experimental Results

Comprehensive computations and simulations of the proposed ensemble classifier have been conducted using the extracted features for detection of finger movement and subsequent classification of the moving directions. The computer simulations were performed using MATLAB 2012b environment on a PC with 64-bit Intel Core i7-2600 CPU @ 3.40 GHz.

**Figure 5:**
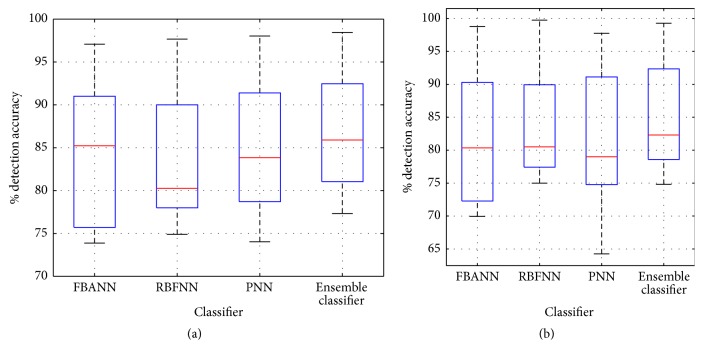
(a) Overall accuracy during detection of resting versus movement for all patients. (b) Classification accuracy while decoding left and right movement activity using base and ensemble classifiers.

**Figure 6:**
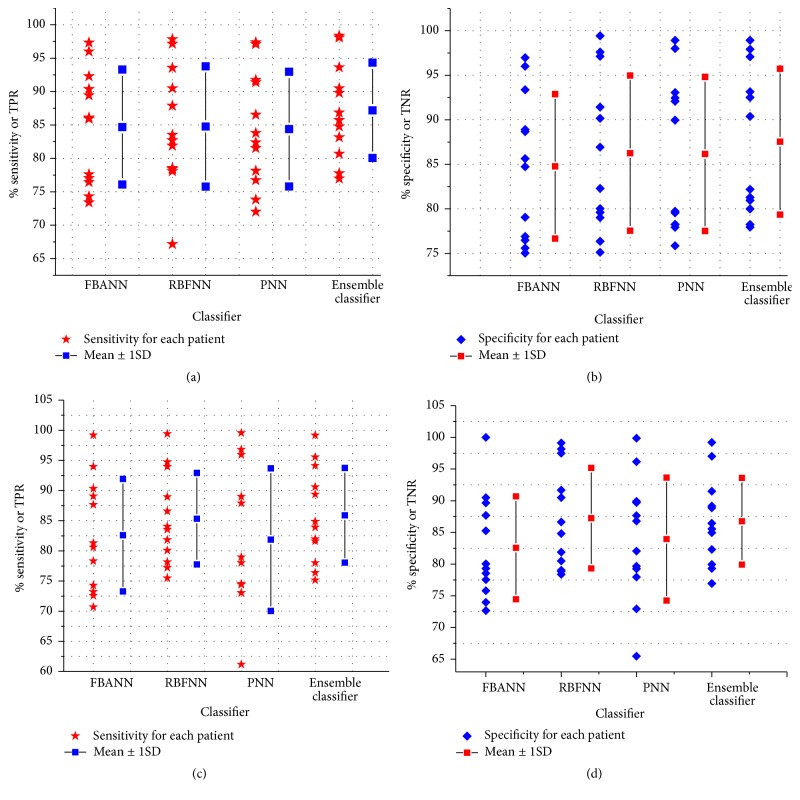
(a) Sensitivity during movement versus resting classification obtained from base and ensemble classifier. (b) Specificity of each base and ensemble classifier and detection of movement and resting activity. (c) Sensitivity during classification of left and right finger clicking events obtained from base and ensemble classifier. (d) Specificity obtained from both base and ensemble classifiers during decoding laterality of movement.

Figures 5(a), 6(a), and 6(b) show the average percentage accuracy, sensitivity, and specificity of the movement decoding for individual patients, respectively. The obtained performance parameters for three base neural networks (mean ± 1SD) are (a) 84.31%  ± 8.56 in accuracy, 84.69%  ± 8.60 in sensitivity, and 84.77%  ± 8.11 in specificity with FBANN; (b) 83.94%  ± 7.99 in accuracy, 84.77%  ± 9.00 in sensitivity, and 86.25%  ± 8.71 in specificity with RBFNN; and (c) 85.03%  ± 8.30 (mean ± 1SD) in accuracy, 84.38%  ± 8.59 in sensitivity, and 86.16%  ± 8.65 in specificity with PNN. With the ensemble classifier, we achieved 87.07%  ± 7.54 in accuracy, 87.19%  ± 7.14 in sensitivity, and 87.54%  ± 8.19 in specificity. These results are about 2–4% better compared to individual base classifier. In addition, from the results in Figures 5(b), 6(c), and 6(d), for laterality of movement decoding, FBANN achieved 82.20%  ± 10.25 in overall accuracy, 82.19%  ± 11.63 in sensitivity, and 82.80%  ± 8.00 in specificity; RBFNN achieved 83.51%  ± 7.84 in overall accuracy, 84.78%  ± 8.93 in sensitivity, and 87.25%  ± 9.41 in specificity; and PNN achieved 81.62%  ± 11.45 in overall accuracy, 81.87%  ± 11.82 in sensitivity, and 83.95%  ± 9.70 in specificity.

The ensemble classifier fused the outputs of three base classifiers (i.e., accuracy in FBANN: 83.042%; in RBFNN: 83.658%; and in PNN: 82.98%) together and achieved 86.073% in detection accuracy, while patients were in resting state and left or right finger movement activity. Therefore, the overall improvement in detection accuracy of resting from left/right finger movement reached about 3.0% (Figure 9(a)) and the overall error rate (OER) of the detection decreased notably. During movement decoding, RBFNN performed better than PNN and FBANN classifiers in terms of accuracy (83.94%  ± 7.99 versus 87.07%  ± 7.54 (*t*(22) = −0.9866, *p* < 0.05)), sensitivity (84.77%  ± 9.00 versus 87.19%  ± 7.14 (*t*(22) = −0.7300, *p* < 0.05)), and specificity (86.25%  ± 8.71 versus 87.54%  ± 8.19 (*t*(22) = −0.3728, *p* < 0.05)). For laterality decoding, RBFNN still managed to achieve better performance than the other two base classifiers in accuracy, sensitivity, and specificity.

Essentially, with various feature set sizes, all the classifiers managed high degree of classification accuracy. RBFNN achieved a lower false positive rate (FPR) but has lower detection rate than PNN classifier in decoding movement. On the other hand, in movement laterality decoding, RBFNN classifier maintained less intra-subject variability in performance than the other two base classifiers. Overall, RBFNN achieved the highest classification rate as well as highest specificity among the three base classifiers. It also performs advantageously in comparison to PNN and FBANN in terms of balanced accuracy (Table 6). Although RBFNN classifier has achieved lowest FPR for both movement and laterality classifications compared to others, it did have higher value of FNR compared to FBANN; more importantly it achieved higher TPR and TNR values than PNN algorithm.

**Figure 7:**
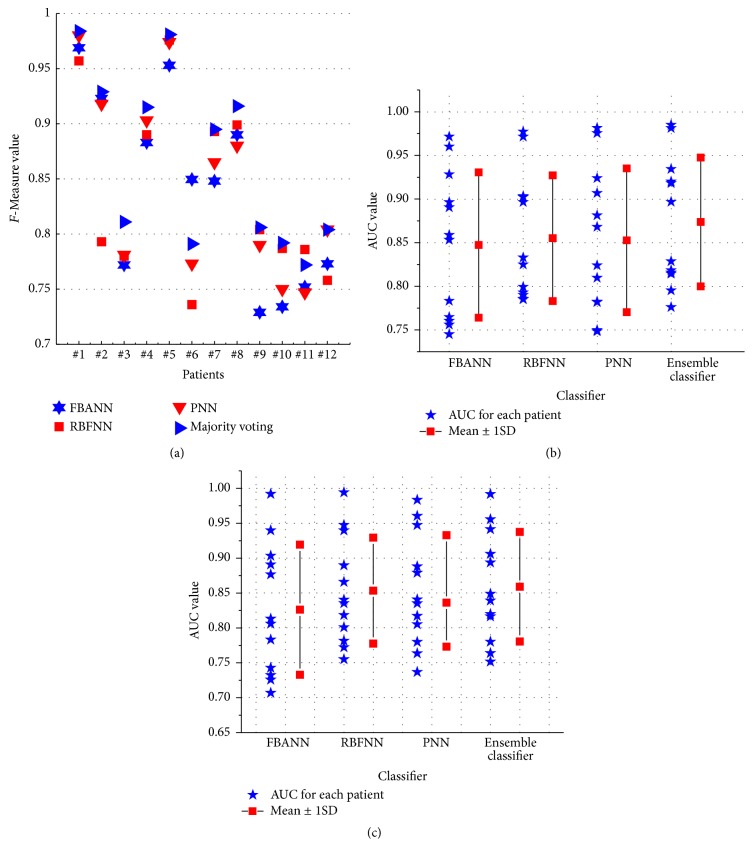
(a) *F*-Measure plot of base and ensemble classifier during movement versus resting classification. (b) Area under the ROC curve (AUC) for each patient with mean ± 1SD obtained from each base and ensemble classifier during movement versus resting classification. (c) Area under the ROC curve (AUC) for each patient with mean ± 1SD obtained from each base and ensemble classifier during left and right finger movement classification.

To show the impact of the imbalanced classes on the performance, we obtained the AUC values for each classifier in movement and laterality decoding as shown in Figures 7(b) and 7(c). It is found that the average value of AUC (0.873) with the ensemble classifier is greater than those with any individual classifier. Similarly, in laterality of movement decoding, the ensemble classifier achieved better AUC (0.859) values than any base classifier.

Although all the base classifiers performed well in detecting movement and its forthcoming laterality, the ensemble classifier based on the majority voting algorithm performed better than the base classifiers in detection, especially in terms of FNR. The FNR rate of the ensemble classifier is improved by 2.98% compared with that of any of the base classifiers in decoding movement versus resting of the patients.

Moreover, we computed other distinctive performance indicators such as *F*-measure (Figure 7(a)), *g*mean-1, and *g*mean-2. Obviously, larger *F*-measure values indicate finer precision and higher sensitivity. *g*-mean value measures the balanced performance of the classifiers between sensitivity, specificity, and precision.

Standardized 1st-order moments of *F*-measure and *g*-mean values for this work are tabulated in Table 5. It is observed from Table 5 that RBFNN performs the best in terms of *F*-measures. In decoding resting versus movement and its forthcoming laterality, RBFNN also shows the highest degree of desirability, since it achieves the highest desirability value among other base classifiers.

From Table 6, it can be seen that PNN and RBFNN classifiers demonstrated better MCC results, showing better agreement between the prediction and actual results in detecting movement and classifying laterality of movement. RBFNN classifier achieves higher BACC value than other base classifiers. However, with the ensemble classifier, the BACC value is improved by at least 2.63% compared to any base classifier in movement and resting classification.

The data from all the patients demonstrated good kappa coefficient values using each classifier while classifying their LFP patterns of movement and laterality (overall values shown in Table 6). The experimental results also showed that the highest kappa value (0.692 ± 0.17) (mean ± 1SD) is obtained using PNN classifier in discriminating movement from resting activity. For movement laterality classification, it managed to have a value of 0.590 ± 0.28 (mean ± 1SD), which indicates a good agreement between actual and predicted identifications.

**Figure 8:**
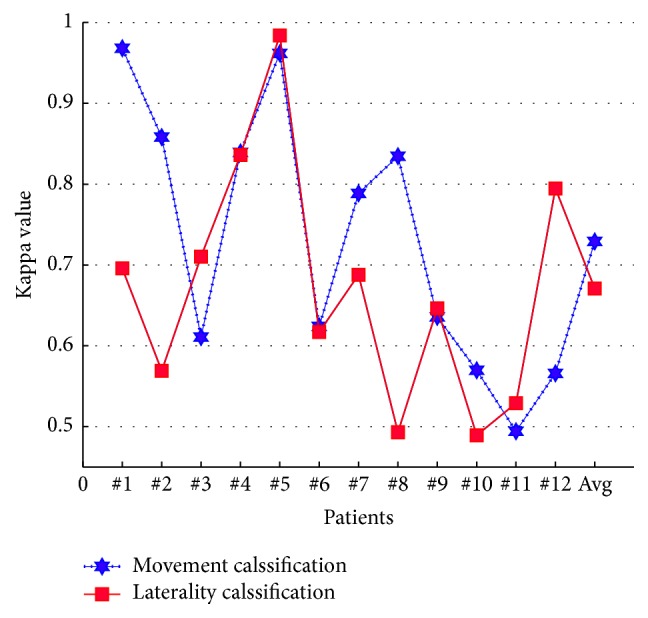
Plot of overall kappa value for movement and its laterality classifications using the ensemble classifier for all participating patients.

Figure 8 shows the kappa values using the ensemble classifier for the datasets generated from all twelve patients. Individual kappa value suitably exceeded 0.4, which is equivalent to an accuracy of >70%. An accuracy of 70% is considered necessary for meaningful communication with a 2-class BCI [42]. Additionally, a very good agreement between the intended and predicted selections (kappa > 0.8 with the peak at 0.96 equivalent to decoding accuracy > 95%) was achieved for two participants while detecting movement and its forthcoming laterality.

Since we do not have enough information such as patient disease severity and handedness, it is difficult to do correlation analysis between movement decoding performance and disease situations. However, based on demographic data, as shown in Table 1, we have computed movement decoding performances, as shown in Table 7. It can be seen that, according to disease types, the patients with Parkinson's disease (PWP) exhibited much higher movement decoding rate than the Dystonia patients. Similarly, the decoding activity of LFP signals recorded through DBS electrodes from STNs achieved higher average accuracy than GPIs.

**Figure 9:**
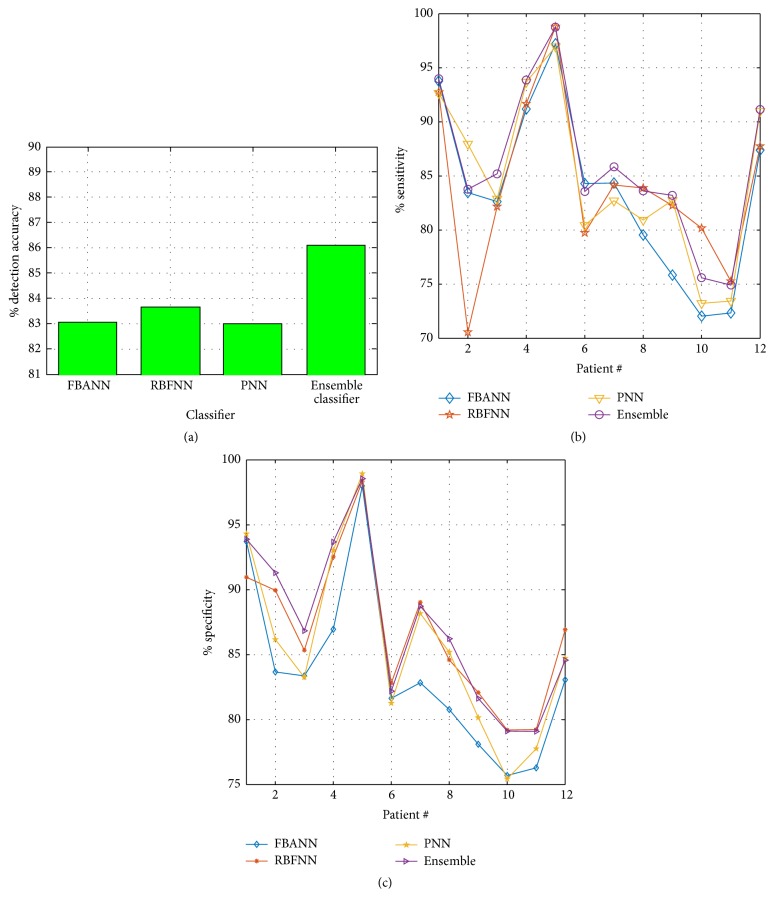
(a) Overall detection accuracy, (b) sensitivity, and (c) specificity of resting, left, and right finger movement activities obtained from base and ensemble classifiers.

To show further robustness of three-class (resting and left or right hand finger movements) classification performance using the ensemble neural network (NN), we have computed numerical performance metrics based on available datasets from 12 PD and Dystonia patients. The results are presented in Figure 9. It can be seen that the ensemble classifier has better performances than any of the base classifiers (accuracy ~3% better than individual classifier). Furthermore, the majority voting also showed greater sensitivity and specificity (Figures 9(b) and 9(c)) as compared to the base classifiers. Other performance measures for both the ensemble and the base classifiers are shown in Table 8.

## 6. Further Discussions

This work investigated the potential advantages of neural network ensemble classifiers for decoding of human finger movements or resting activity using deep brain local potential signals. The aforementioned testing results show that the average decoding performance during movement and its laterality decoding process using the proposed ensemble classifier is very promising and this methodological framework may lead to the development of more effective BMI applications. With various feature set sizes, it was demonstrated that RBFNN has been proven to be better decoder by managing impressive overall classification rate (CR) and PNN has shown the worst performance among the three weak learners. The RBFNN classifier performs advantageously over PNN and FBANN in terms of balanced accuracy with the lowest false detection rate. However, a few factors could have degraded the classifiers' performance; they are the unbalanced number of trials in the dataset, the unbalanced variability within the classes, the higher redundancy, and the unbalanced variation in the feature sets. Further additional factors need to be considered such as magnitude variation among the frequency bands as limited or less expertise of the participants to execute action according to stimulus applied, motivation and concentration to respond, and patient insensitiveness due to fatigue, age, and patient's depth of diseases.

(i) The total number of trials for the clicking events taken from each patient showed potential variation of decoding performance [43]. In the experimental session, obtained LFP datasets were limited in size. 114 ± 43.6 trials (mean ± 1SD) consisted of minimum 56 and maximum 202 trials across all subjects employed in the movement decoding process. For most of the patients, the number of trials is unbalanced for each class. The average number of trials of each class is 58.2 ± 23.6 (mean ± 1SD) with a minimum of 25 trials and maximum of 113 trials. This unbalancedness of the trials may contribute to the increase of the overall error rate (OER) for some participants in decoding. However, recent researches also suggest that a larger number of trials are needed to more accurately and robustly assess the predictive model [42].

(ii) It can be seen from Figures 6(c) and 6(d) that patients rapidly and efficiently responded during visually cued right hand finger clicking events compared to left finger clicking events for both STN's and GPI's LFP signal (overall specificity: 87.07%  ± 7.21; overall sensitivity: 84.86%  ± 9.54 (mean ± 1SD)). Although we had no abundant information about handedness of the patients, it can be considered that right handed patients were better trained than left handed patients due to generality of right handedness among human inhabitants.

(iii) LFPs obtained from the patients are more stable than single-neuron activity or noninvasive EEG; nonetheless it can contaminate with conspicuous motion artifact and patient insensitiveness due to fatigue, which ultimately deteriorates LFP signal momentarily during onset of movement event. The proposed decoder system has shown its effectiveness by addressing the aforementioned limitation to a greater extent by using different types and range of patients.

(iv) Although two-session recordings were obtained from four of the participants, LFP recordings from each patient used in this study are involved in a single session only. As a future work, several sessions will be recorded and single-session features will be enforced as a test set, while remaining sessions will be used to train the intelligent classifier to decide substantial, stable, and trustworthy decoding outcome. However, we will carry out further research on early prediction of movement conducted by normal and abnormal people in a controlled and distraction-free environment that is applicable in widespread neuro-interface scenario. With consideration of the above limitations, the performance of the proposed ensemble classifier in LFP movement detection and classification is very encouraging. To the best of our knowledge, these results achieved in this work are better than those reported previously in the literature in terms of detection rate and the number of patients [1]. Theoretically, the proposed two-layer two-class classifier could be replaced with a three-class classifier. However, our results showed that two-class classifiers are more robust for the datasets used in this work.

## 7. Conclusion

This study explores an innovative neural network ensemble classifier for effective identification of voluntary movements extracted from oscillatory activity of LFP signals recorded bilaterally in the STN or GPI of twelve Parkinson's disease and Dystonia patients. A majority voting algorithm is used in the ensemble classifier to fuse the results from three individual neural network classifiers. The experimental results demonstrate that decoding rate of clicking events is greater than its laterality of clicking (87.07%  ± 7.54 versus 85.41%  ± 8.68 (mean ± 1SD)) using the ensemble neural network classifier. The performances of movement decoding for each base classifier were investigated and evaluated and it is found that the ensemble classifier is consistently better than the base classifiers or other similar classifiers in terms of convergence rate as well as classification accuracy. The results also demonstrated that PNN achieves the best detection accuracy (DA) (85.03%  ± 8.30 (mean ± 1SD)) among those three base classifiers in identifying event. In predicting sequential clicking events, RBFNN (83.51%  ± 7.84 (mean ± 1SD)) outperforms FBANN and PNN. The proposed optimal classifying system may provide a channel for developing wearable and wireless smart stimulation devices that can predict involuntary movements (such as tremor) and adaptively respond to the onset of abnormal neurological events. With three different neural networks as the base classifiers, the classification performance improvement of the ensemble classifier appeared to be modest and yet noticeable. However, ensemble classifier was demonstrated to be an effective approach to improving human finger movement decoding and interpretation performance. It should be pointed out that the real-time convergence is a very important issue for any classification algorithm; however, the investigation of the proposed ensemble classifier is limited to offline analysis at this stage. Our future work in this area includes improvement of better feature extraction algorithms and the optimization of the base classifiers for the ensemble classifier.

## Figures and Tables

**Algorithm 1:**
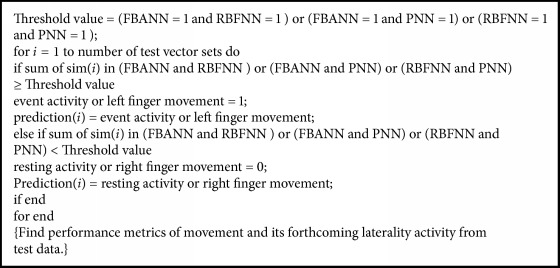
Decision-based pseudocode for decoding event or resting and left or right finger movement activities.

**Table 1:** Recording and clinical details of patients.

Patient #	Age	Sex	Years in disease	PD or Dystonia	Elec. placed	Electrode pair used
1	58	F	10	PD	STN	L23/R12
2	63	F	3	PD	STN	L12/R12
3	59	M	7	PD	STN	L01/R01
4	60	M	13	PD	STN	L12/R01
5	72	F	21	PD	GPI	L01/R01
6	55	M	10	PD	STN	L12/R01
7	36	M	14	Dystonia	GPI	L12/R12
8	53	M	5	Dystonia	GPI	L01/R01
9	23	M	7	Dystonia	GPI	L12/R01
10	54	F	38	Dystonia	GPI	L01/R01
11	40	M	25	Dystonia	GPI	L01/R01
12	32	F	24	Dystonia	GPI	L12/R23

**Table 2:** The distribution of trials used for each patient.

Patient	Number of trials used		
	Left finger movement	Right finger movement	Total
1	52	41	93
2	31	37	68
3	71	84	155
4	31	82	113
5	56	54	110
6	25	31	56
7	61	62	123
8	73	72	145
9	34	28	62
10	59	48	107
11	113	89	202
12	80	76	156

**Table 3:** Threshold settings for individual classifier while detecting the movement and resting activity.

Class	Base classifier threshold setting	Classifier final output after threshold setting
Event condition	≥0.5	1
Resting condition	<0.5	0
Left movement	≥0.5	1
Right movement	<0.5	0

**Table 4:** Statistical performance measures for decoding of movement and its laterality activity.

Overall accuracy	Sensitivity or DR	Specificity	Overall error rate (OER)
$\frac{TP+TN}{TP+TN+FP+FN}$	$\frac{TP}{TP+FN}$	$\frac{TN}{TN+FP}$	$\frac{FP+FN}{TP+TN+FP+FN}$

**Table 5:** Standardized 1st-order moment of evaluation measures.

Classifiers evaluated	*F*-Measure	*g*mean-1	*g*mean-2	Desirability value
During decoding movement and resting				
FBANN	9.77	9.92	10.17	9.13
RBFNN	10.41	11.52	11.74	10.25
PNN	10.08	10.57	10.32	10.02
Ensemble	11.08	11.78	11.82	11.22

For decoding left and right finger movement				
FBANN	7.27	8.09	8.62	8.25
RBFNN	8.37	11.30	11.90	9.39
PNN	6.88	7.96	8.15	7.58
Ensemble	8.46	9.58	10.98	9.67

**Table 6:** Statistical significance measures of the classifiers while decoding movement from the resting.

Classifiers evaluated	MCC value	Overall kappa value, $\bar{k}\pm \varphi (k)$	BACC
For decoding movement versus resting			
FBANN	0.691	0.607 ± 0.25	84.73
RBFNN	0.691	0.599 ± 0.27	85.51
PNN	0.701	0.692 ± 0.17	85.27
Ensemble	0.737	0.729 ± 0.16	87.36

For decoding left versus right finger movement			
FBANN	0.647	0.634 ± 0.20	82.49
RBFNN	0.665	0.563 ± 0.23	85.96
PNN	0.636	0.590 ± 0.28	82.91
Ensemble	0.712	0.671 ± 0.14	85.96

**Table 7:** Comparison of detection performances of the ensemble classifier for disease conditions and groups of patients.

Patient groups/LFP signal collection methods	Overall accuracy (%)	TPR (%)	TNR (%)
Patients with PD	88.79	88.17	92.33
Patients with Dystonia	82.03	81.55	81.81
LFPs from STNs	89.14	86.56	89.87
LFPs from GPIs	84.62	83.95	84.55

**Table 8:** Statistical performance measures of base and ensemble classifiers to classify resting and left or right finger movement.

Classifiers	*F*-Measure	AUC	Kappa	False positive rate (FPR)
FBANN	0.8277	83.67	0.6154	16.28
PNN	0.8433	85.29	0.6687	13.95
RBFNN	0.8365	85.42	0.5905	13.26
*Ensemble *	*0.8574*	*86.63*	*0.7067*	*12.88*
